# Unexpected renal influence on isavuconazole trough concentrations: two case reports and literature review

**DOI:** 10.3389/fmed.2025.1625697

**Published:** 2025-08-19

**Authors:** Hui Li, Xiaoshuang He, Qiuya Lu, Ling Wang, Jieling Jiang, Wenhui Gao, Lining Wang, Jiong Hu, Jie Fang, Xiaolan Bian

**Affiliations:** ^1^Department of Pharmacy, Ruijin Hospital, Shanghai Jiaotong University School of Medicine, Shanghai, China; ^2^Department of Laboratory Medicine, Ruijin Hospital, Shanghai Jiaotong University School of Medicine, Shanghai, China; ^3^Shanghai Institute of Hematology, Department of Hematology, Blood & Marrow Transplantation Center, Collaborative Innovation Center of Hematology, Ruijin Hospital, Shanghai Jiaotong University School of Medicine, Shanghai, China

**Keywords:** invasive aspergillosis, isavuconazole, therapeutic drug monitoring, renal function, adverse drug reactions

## Abstract

Isavuconazole (ISA) is a first-line treatment for invasive aspergillosis, with routine therapeutic drug monitoring (TDM) typically deemed unnecessary. Although the drug label and prior studies suggest renal function does not affect ISA pharmacokinetics, our findings in two high-risk patients challenge this perspective, showing an inverse correlation between ISA trough concentrations and estimated glomerular filtration rate (eGFR). Trough concentrations exceeding the reported toxicity threshold were associated with adverse drug reactions (ADRs). Given the limited sample size, large-scale retrospective and prospective studies are urgently needed to confirm the impact of renal function on ISA and to develop individualized TDM strategies that optimize efficacy and minimize toxicity.

## Introduction

ISA is a second-generation triazole antifungal, available in oral and intravenous formulations, approved for treating invasive aspergillosis (IA) and mucormycosis in adults (1). It offers broad-spectrum activity against yeasts, dimorphic fungi, and molds, with predictable pharmacokinetics and minimal drug interactions (2). Administered as the water-soluble prodrug isavuconazonium sulfate, it is rapidly converted to ISA, primarily metabolized by the cytochrome P450 enzymes (CYP3A4/5), with a half-life of approximately 130 h. Unlike other azoles, its *β*-cyclodextrin-free intravenous formulation minimizes nephrotoxicity, favoring use in renal impairment. Drug labeling and a Phase 1 trial indicate ISA’s pharmacokinetics are unaffected by renal function, requiring no dose adjustments in renal impairment and end-stage renal disease (ESRD) (3). A VITAL study population pharmacokinetic analysis demonstrated no impact of eGFR on ISA clearance (4). However, we observed elevated ISA trough concentrations associated with renal impairment in two patients, causing adverse drug reactions (ADRs). To our knowledge, this is a previously unreported observation that suggests renal function may influence ISA pharmacokinetics, warranting further investigation.

## Case description

### Case 1

An 82-year-old man with hypertension, type 2 diabetes mellitus, and prior atrial flutter ablation was admitted to the ICU on 7 October 2024, after 3 h of unconsciousness. He was comatose with a blood pressure of 157/68 mmHg. Laboratory results showed an elevated white blood cell count of 12.72 × 10^9/L (neutrophils 83.9%), hemoglobin of 109 g/L, platelet count of 215 × 10^9/L, and eGFR of 93.2 mL/min/1.73 m^2^. Head CT revealed a cerebral hemorrhage, managed conservatively with neuroprotection and control of blood pressure and other underlying diseases.

A chest CT showed bilateral pulmonary opacities with consolidation in the right lower lobe, suggesting infection. Empirical meropenem and vancomycin were initiated for presumed pneumonia. On day 4, sputum culture identified carbapenem-sensitive *Escherichia coli (E. coli)*, and eGFR decreased to 54.6 mL/min/1.73m^2^, leading to vancomycin discontinuation. By day 9, *E. coli* was cleared (white blood cell count normalized to 4.78 × 10^9^/L), and meropenem was discontinued. However, sputum next-generation sequencing (NGS) detected *Aspergillus fumigatus*, confirming invasive pulmonary aspergillosis. The treatment was changed to intravenous ISA with a loading dose of 200 mg every 8 h for 48 h (6 doses), followed by a maintenance dose of 200 mg once daily, combined with nebulized amphotericin B. The eGFR further decreased to 43.8 mL/min/1.73m^2^, with stable hemoglobin (105 g/L), platelet count (200 × 10^9^/L), alanine aminotransferase (ALT, 19 U/L), and aspartate aminotransferase (AST, 22 U/L).

**Figure 1:**
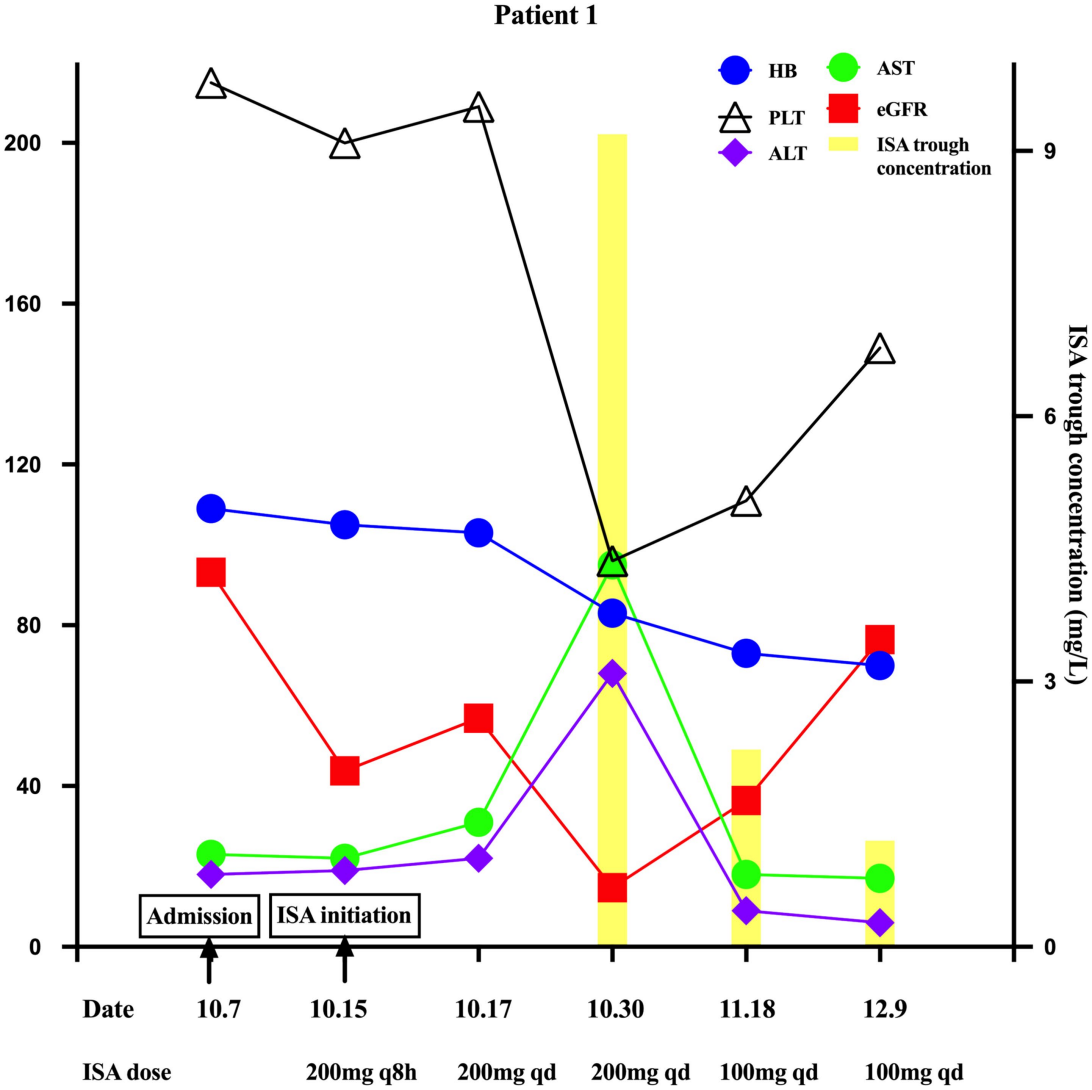
Key changes in ISA trough concentration, eGFR, hemoglobin, platelet count, ALT, and AST in Patient 1 during ISA therapy with varying renal function.

On day 24, the patient developed severe renal dysfunction (eGFR 14.7 mL/min/1.73m^2^), decreased hemoglobin (83 g/L) and platelet count (96 × 10^9^/L), and elevated liver enzymes (ALT 68 U/L, AST 95 U/L). Other liver function parameters remained normal. Following the exclusion of disease-related factors and non-ISA medication effects, these mild ADRs were attributed to ISA toxicity. The Naranjo Adverse Drug Reaction Probability scale yielded a score of 9, indicating a definite causal relationship. TDM revealed an elevated ISA trough concentration of 9.19 mg/L, likely due to reduced renal clearance and the cause of the ADRs. The TDM-guided ISA dose was reduced to 100 mg daily. On day 42, eGFR improved to 36.4 mL/min/1.73m^2^, ISA trough concentration decreased to 2.23 mg/L, hemoglobin decreased to 73 g/L, platelet count increased to 111 × 10^9^/L, and liver enzymes were normalized (ALT 9 U/L, AST 18 U/L). A follow-up chest CT showed a reduction in the size of the lesion; nebulized amphotericin B was discontinued, and the ISA dose was maintained. On day 63, eGFR increased to 76.4 mL/min/1.73m^2^, ISA trough concentrations decreased to 1.20 mg/L, platelet counts normalized to 149 × 10^9^/L, and hemoglobin stabilized at 70 g/L. Key clinical and laboratory changes are illustrated in Figure 1.

### Case 2

A 61-year-old man with atrial fibrillation and chronic myeloid leukemia (diagnosed in March 2022) progressed to acute lymphoblastic leukemia after developing resistance to first-, second-, and third-generation tyrosine kinase inhibitors. He underwent allogeneic hematopoietic stem cell transplantation in September 2023. Persistent chronic graft-versus-host disease (GVHD), with recurrent ascites and pleural effusion, was poorly controlled with long-term corticosteroids and immunosuppressants, requiring voriconazole, entecavir, letermovir, and valganciclovir for fungal and viral prophylaxis.

**Figure 2:**
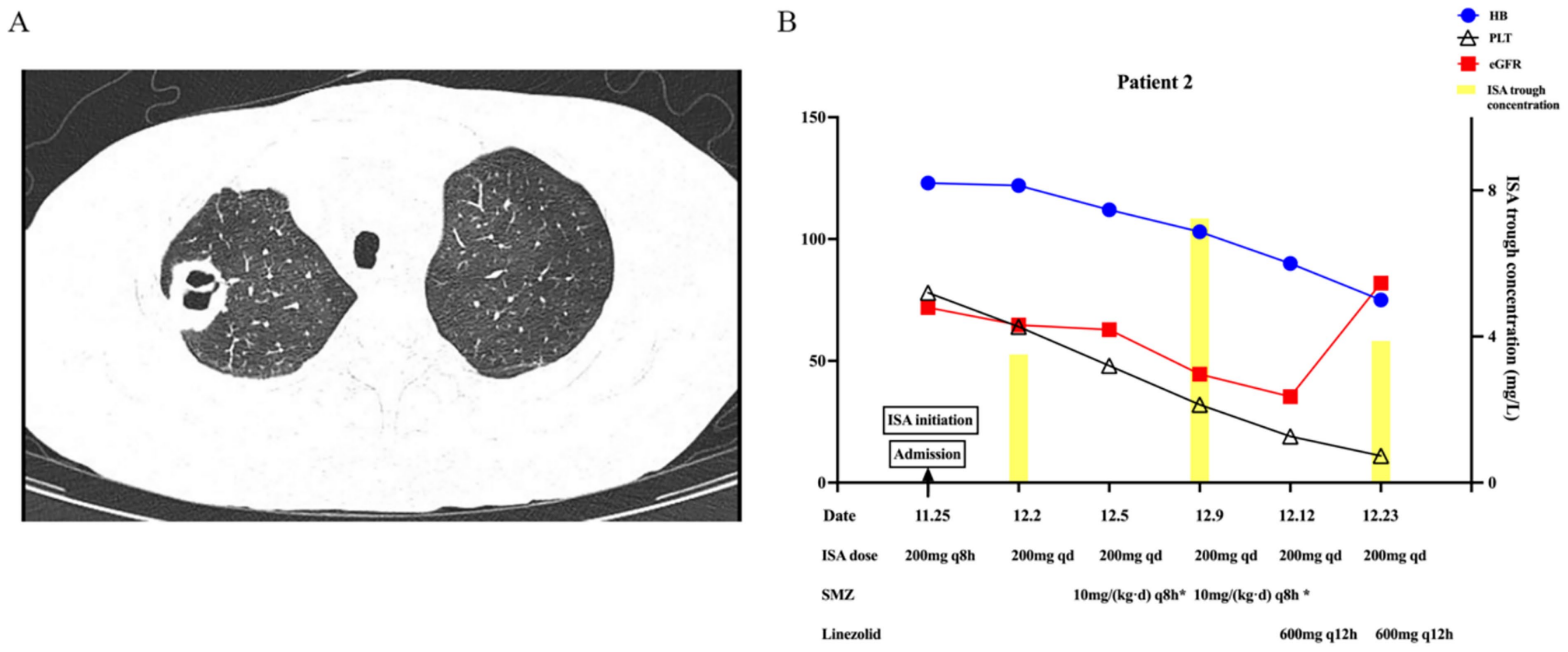
Imaging and key changes in Patient 2 during ISA therapy. **(A)** Imaging findings of pulmonary cavitary lesion. **(B)** Key changes in ISA trough concentration, hemoglobin, and platelet count with varying renal function.

On 18 November 2024, an outpatient chest CT scan revealed a 12-mm cavitary nodule in the right upper lobe (Figure 2). As a fungal infection could not be ruled out, voriconazole was discontinued, and treatment was switched to oral ISA with a loading dose of 200 mg every 8 h for 48 h (six doses), followed by a maintenance dose of 200 mg once daily, combined with intravenous caspofungin upon the patient’s admission on 25th November. The initial laboratory results showed an eGFR of 71.9 mL/min/1.73m^2^, hemoglobin of 123 g/L, and a platelet count of 78 × 10^9^/L. On 2nd December, the eGFR was 64.7 mL/min/1.73m^2^, ISA trough concentration was 3.51 mg/L, hemoglobin was stabilized at 122 g/L, and platelet count decreased to 64 × 10^9^/L. On 5th December, bronchoalveolar lavage fluid (BALF) with NGS identified *Nocardia*, prompting the initiation of sulfamethoxazole (SMZ) and caspofungin discontinuation. On 9th December, eGFR decreased to 44.5 mL/min/1.73m^2^, correlating with ISA trough concentration rise to 7.23 mg/L, while hemoglobin (103 g/L) and platelet counts (32 × 10^9^/L) declined. After ruling out disease progression, these moderate ADRs were more likely attributed to SMZ toxicity, though ISA toxicity could not be excluded. The Naranjo Scale score of 3 only indicated a probable causal relationship between ISA and these ADRs. Considering the ADRs of renal impairment and reduction of hemoglobin and platelets, SMZ was adjusted to linezolid combined with amoxicillin-clavulanate on 12th December. On 23rd December, eGFR improved to 81.9 mL/min/1.73m^2^, and ISA concentration decreased to 3.88 mg/L, but the worsening platelet count (11 × 10^9^/L) was primarily attributed to linezolid. A chest CT showed reduced cavitation, prompting a switch to contezolid for *Nocardia* treatment. By 14 January 2025, platelet counts had increased to 33 × 10^9^/L, and CT showed decreased cavitation. Figure 2 summarizes the significant changes.

## Discussion

Invasive fungal infections (IFIs), including IA, pose a significant mortality risk for immunocompromised patients (undergoing transplantation or corticosteroid use) and ICU patients. Case 1 (ICU patient) and Case 2 (HSCT recipient with GVHD) validate this vulnerability. A global observational study reported that 16% of ICU infections involved fungi, with a higher mortality risk (5). HSCT patients have a 16% IFI incidence, with GVHD being a major risk factor (6). Consistent with these findings, Case 2, experiencing GVHD, increased IFI risk, underscoring the need for infection monitoring.

ISA and voriconazole are guideline-recommended first-line treatments for suspected IA (7, 8). Clinical trials demonstrate that ISA’s efficacy is non-inferior to voriconazole for invasive mold infections and comparable to caspofungin and fluconazole for invasive candidiasis, and equivalent to amphotericin B for mucormycosis, with favorable tolerability (9–11). Prospective studies have explored ISA for primary prophylaxis (12–14). Its intravenous formulation prevents nephrotoxicity, and its predictable pharmacokinetics and improved tolerability make it preferable to voriconazole (2, 15). Both patients received ISA for suspected IFIs and tolerated it despite ADRs linked to elevated trough concentrations.

Guidelines and experts do not recommend routine TDM for ISA due to its predictable pharmacokinetics, but suggest it for specific groups such as children or patients on extracorporeal membrane oxygenation, monitoring trough concentration over AUC/MIC TDM (16–18). In our study, trough concentration blood samples were collected within 30 min prior to the next scheduled dose under steady-state conditions, in accordance with recommendations, and measured using a validated liquid chromatography-tandem spectrometry (LC–MS/MS) method. Although the optimal therapeutic ISA trough concentration range remains undefined, A study of 264 trough concentrations from 19 patients suggested a therapeutic window of 2–5 mg/L, with 5.13 mg/L as the toxicity threshold (80% sensitivity, 72.3% specificity) (19). However, these findings are limited by the small sample size and reliance on trough concentrations alone, necessitating a further large cohort with additional PK parameters, such as AUC. Both patients in this report developed ADRs at trough concentrations exceeding this threshold. In Case 1, a trough of 9.19 mg/L resulted in a marked reduction in hemoglobin and platelet count, as well as minor hepatocellular injury; lowering the dose to 100 mg/day reduced the concentration to 2.23 mg/L, significantly reducing ADRs (Figure 1). In Case 2, severe reductions in hemoglobin and platelet counts are more likely attributed to SMZ and linezolid; however, a synergistic effect of an elevated ISA concentration (7.23 mg/L) could not be excluded. In the phase 3 SCURE trial, hematologic, renal, and hepatobiliary disorders occurred in 30, 21, and 9% of patients (9).

Literature reports a wide range of ISA trough concentrations (0.24–15.4 mg/L), with median values of 1.36 to 4.47 mg/L and inter-individual variability of 28.2–54.8% (Table 1). Intra-individual variability spans 30.7–43.4% providing a benchmark for assessing case-specific deviations. Notably, studies suggest renal function does not affect ISA pharmacokinetics. A study in solid-organ transplant recipients undergoing continuous RRT (CRRT) found transmembrane clearance accounted for only 0.7% of total ISA clearance, indicating minimal CRRT removal and no need for dose adjustment (20). A phase 1 trial comparing healthy controls and patients with mild, moderate, and severe renal impairment found no significant differences in maximum concentration or area under the curve (Table 1), but measured non-trough metrics, unlike guideline-recommended trough monitoring (3, 17). In contrast, our cases suggest renal function influences ISA trough concentration. In Case 1, as eGFR increased from 14.7 to 76.4 mL/min/1.73m^2^, ISA trough concentration decreased from 9.19 to 1.2 mg/L (86.9% variability). In case 2, as eGFR decreased from 71.9 to 44.5 mL/min/1.73m^2^, ISA trough concentration increased from 3.51 to 7.23 mg/L (106% variability). This inter-individual variability observed in our cases (86.9 and 106%) exceeds the upper limit of previously reported ranges (28.2–54.8%), suggesting a potential influence of renal impairment on ISA trough concentration variability. This variability is unlikely due to other known factors, particularly drug–drug interactions, as neither patient was concurrently receiving CYP3A4/5 inducers or inhibitors. Although this finding appears inconsistent with the minimal renal clearance characteristic of ISA, the existing literature indicates that both chronic and acute renal impairment may alter CYP enzyme activity (21, 22). In acute kidney injury, the clearance of non-renally eliminated drugs, such as vancomycin and imipenem, is also affected (22). Elevated levels of parathyroid hormone, cytokines, and uremic toxins in the context of renal dysfunction may suppress CYP activity, Phase II metabolic reactions, and drug transport processes. Consequently, we hypothesize that the effect of renal impairment on ISA trough concentration is primarily due to altered metabolism—particularly reduced CYP activity—rather than impaired excretion. Given the small size, larger trials are needed to validate these findings and further elucidate the underlying mechanisms.

**Table 1:** ISA TDM pharmacokinetic parameters reported in the literature.

Reference	No. of patient	study design	Subject characteristic	Dose regimen	Pharmacokinetic Parameters
Townsend et al. (3)	49	A phase 1 open-label single-dose parallel group study	Part1: healthy controls (*n* = 9), mild RI (*n* = 8), moderate RI (*n* = 8), severe RI (*n* = 5); Part 2: healthy controls (*n* = 8), ESRD (*n* = 11)	A single intravenous dose of 200 mg ISA; ESRD patients received an additional dose t prior to dialysis (day 15)	1. healthy controls vs. mild RI vs. moderate RI vs. severe RI 1) C_max_: 4.4 ± 0.7 vs. 3.9 ± 1.1 vs. 4.1 ± 1.4 vs. 3.4 ± 0.9 2) AUC_∞_:98.8 ± 50.5 vs. 96.2 ± 46.9 vs. 97.2 ± 26.3 vs. 98.8 ± 53.9 3 2. healthy controls vs. ESRD day 1 vs. ESRD day 15 1) C_max_: 4.6 ± 1.1 vs. 3.7 ± 1.3 vs. 3.7 ± 0.8 2) AUC_∞_: 94.7 ± 32.2 vs. 95.7 ± 78.6 vs. -
Bose et al. (14)	65	An open-label, prospective, phase 2 study	AML/MDS patients who become neutropenic as a result of their first RIC and use ISA as primary antifungal prophylaxis	200 mg ISA every 8 h for six doses (48 h) and, thereafter, 200 mg once daily	1) C_min_ (day 1): 3.37(1.81–7.65); 2) C_min_ (day 15):3.95 (1.56–9.25) 3) C_min_ change (days 1–15): 2 patients >3
Bolcato et al. (23)	33	Monocentric retrospective study	An adult patient who is treated with ISA with at least one ISA C_min_	200 mg ISA every 8 h for six doses (48 h) and, thereafter, 200 mg once daily	1) C_min_: 2.8 (IQR 2.0–3.7) 2) Inter-individual variability: 41.5% 3) Intra-individual variability: 30.7%
Fernández Ledesma et al. (18)	15	Retrospective observational study	Pediatric patients (≤18 years) who received intravenous or oral ISA for IFI treatment	Patients weight ≤35 kg, initial doses of 5.4 mg/kg/day (up to a maximum of 200 mg); Patients weight >35 kg, initial doses of 200 mg/day. A loading dose every 8 h during the first 48 h, followed by a maintenance dose once daily.	1) C_min_: 3.1 (IQR 2.4–4.5) 2) Outside therapeutic range: 46.8% 3) Subtherapeutic(<2.5): 30.6% 4) Supratherapeutic(>5): 16.2%
Bertram et al. (24)	62	Not mentioned	Adult patient with or without COVID-19 who suffered from invasive aspergillosis or other invasive fungal infections have been treated with ISA in the ICU	200 mg, 400 mg, or 600 mg ISA once daily	1) C_min_: 1.64 (0.24–5.67) 2) C_min_ < 1: 34.4% RRT(yes) vs. RRT(no) 3) C_min_ (95%CI): 1.78 (1.56–2) vs. 1.36(1.13–1.58) 4) C_min_ < 1: 30.2% vs. 42.6%
Furfaro et al. (19)	19	A single-center retrospective study	Adult patients received ISA who survived>4 days and had at least one sample sent for TDM	200 mg ISA every 8 h for six doses (48 h) and, thereafter, 200 mg once daily	1) C_min_: 4.47(0.52–10.9) 2) Inter-individual variability: 28.2% 3) Intra-individual variability: 36.6%
Hohl et al. (25)	41	A retrospective study	adult patients with IA or other fungal infections who have been treated with ISA and routine TDM.	200 mg ISA every 8 h for 6 doses (48 h) and, thereafter, 200 mg once daily	1) C_max_: 2.36 (0.41–7.79) 2) C_min_: 1.74 (0.24–4.96) 3) C_min_ < 1 mg/L: 31.7%
Risum et al. (26)	35	A retrospective study	Adult patients received ISA and TDM during the study period.	24 patients received a loading dose. The maintenance dose was different, ranging from 150 mg to 600 mg per day.	1) C_min_: 4.3 (0.5–15.4). 2) Inter-individual variability: 54.8% 3) Intra-individual variability: 43.4%
Biagi et al. (20)	7	Not mentioned	Adult solid-organ transplant patients undergoing continuous renal replacement therapy,	200 mg ISA every 8 h for six doses (48 h) and, thereafter, 200 mg once daily	1) C_max_: 4.0 ± 1.46 2) C_min_: 1.76 ± 0.7 3) AUC:54.01 ± 20.98 mg/L

This case series is limited by its small sample of two patients. Although the drug label and phase 1 trial (*n* = 8 mild, *n* = 8 moderate, *n* = 8 severe renal impairment, *n* = 11 ESRD) suggest that renal function does not influence ISA concentration (3), our findings indicate that impaired renal function is associated with higher trough concentrations compared to normal renal function. Therefore, we recommend that clinicians and pharmacists consider TDM and closely monitor for ADRs in patients with renal impairment receiving ISA to guide dose adjustments and minimize the risk of toxicity. To confirm our preliminary observations, retrospective large-sample studies are needed to further validate the impact of renal function on the PK of ISA. Subsequently, prospective multicenter cohort studies should be initiated to develop individualized dosage strategies based on renal function, thereby optimizing therapeutic efficacy and ensuring safety.

## Data Availability

The original contributions presented in the study are included in the article/supplementary material, further inquiries can be directed to the corresponding authors.
